# Seasonal Patterns of Preterm Birth During the COVID-19 Pandemic: A Retrospective Cohort Study in Romania

**DOI:** 10.3390/medicina61081398

**Published:** 2025-08-01

**Authors:** Paula Trif, Cristian Sava, Diana Mudura, Boris W. Kramer, Radu Galiș, Maria Livia Ognean, Alin Iuhas, Claudia Maria Jurca

**Affiliations:** 1 Doctoral School of Biomedical Sciences, University of Oradea, 410087 Oradea, Romania; 2 Department of Preclinical Disciplines, Faculty of Medicine and Pharmacy, University of Oradea, 410087 Oradea, Romania; 3 Department of Pediatrics, Emergency County Hospital Bihor, 410167 Oradea, Romania; 4 Department of Medical Sciences, Faculty of Medicine and Pharmacy, University of Oradea, 410087 Oradea, Romania; 5 Department of Neonatology, Emergency County Hospital Bihor, 410167 Oradea, Romania; 6 Department of Neonatology, Poznan University of Medical Sciences, 61-701 Poznan, Poland; 7 Department of Neonatology, Clinical County Emergency Hospital, 550245 Sibiu, Romania; 8 Faculty of Medicine, Lucian Blaga University, 550024 Sibiu, Romania; 9 Regional Center of Medical Genetics Bihor, County Emergency Clinical Hospital Oradea (Part of ERN ITHACA), 410469 Oradea, Romania

**Keywords:** COVID-19, preterm birth, maternal-fetal medicine, stillbirth, high-risk pregnancy, perinatal pathology, Romania

## Abstract

*Background and Objectives:* Preterm birth and stillbirth are primary adverse pregnancy outcomes. Research during the COVID-19 pandemic revealed reductions in preterm birth in some countries, while stillbirth rates increased or remained unchanged. These findings suggest the presence of preventable risk factors associated with changes in physical activity and lower exposure to community-acquired infections due to lockdown measures, altered social interaction patterns or reduced access to antenatal care. Assessing seasonal variation may offer insights into whether lifestyle changes during the COVID-19 lockdown period influenced preterm birth rates. *Materials and Methods*: This retrospective cohort study used data from the electronic medical records of Bihor and Sibiu counties. Preterm deliveries (<37 weeks) and stillbirths during the COVID-19 pandemic (2020 and 2021) were compared with the corresponding pre-pandemic (2018 and 2019) and post-pandemic (2022 and 2023) period. Preterm birth rates during summer and winter in the pre-pandemic, pandemic, and post-pandemic years were analyzed. A comparison with rates during strict lockdown was made. *Results*: Out of 52,021 newborn infants, 4473 were born preterm. Preterm birth rates remained stable across all three periods (*p* = 0.13), and no significant seasonal pattern was identified (*p* = 0.65). In contrast, stillbirth rates increased notably during the strict lockdown period, with the median incidence almost doubling compared to other periods (0.87%, *p* = 0.05), while remaining unchanged during the rest of the pandemic (*p* = 0.52). *Conclusions*: Our study found that preterm birth rates remained unaffected by the pandemic and lockdown periods, while stillbirths increased significantly during the strict lockdown. These findings highlight the importance of maintaining access to timely antenatal care during public health emergencies to prevent adverse perinatal outcomes.

## 1. Introduction

About 45% of child deaths under five years of age are newborns (2.7 million out of 5.9 million in 2014), with 60–80% of these being premature or small for gestational age [1,2]. Preterm infants face a significantly higher risk of mortality compared to infants born full-term. They are especially vulnerable to a range of health issues, including impaired respiration, feeding difficulties, and growth failure. Additionally, preterm infants are more likely to experience developmental disabilities, such as cerebral palsy, retinopathy of prematurity, or long-term consequences such as chronic lung disease [3,4,5,6]. Given the considerable burden of preterm birth, understanding its causes and risk factors is essential for improving neonatal outcomes.

Preterm labor is now acknowledged as a syndrome initiated through multiple mechanisms, including inflammation or infection. Numerous maternal and fetal characteristics are linked to preterm birth, including maternal demographic factors, pregnancy history, current pregnancy characteristics, harmful behaviors, infections, and various biological and genetic markers [7,8].

While biological and clinical aspects are crucial, another key challenge is the impact of preterm birth on healthcare systems. Neonatal intensive care medicine is expensive and personnel-intensive. The allocation of resources is very cumbersome since the rate of preterm births is not constant over time. Periods of low occupancy in the NICU are followed by periods of high occupancy, and then resources are strained. The ratio of nurses to preterm infants has an effect on outcomes such as infections or in-hospital mortality [9,10,11]. Therefore, the allocation of resources has both economic and medical implications.

One important aspect that influences both clinical outcomes and healthcare burden is the observed temporal pattern of preterm births. The seasonality of preterm birth has been well documented [7,8,12,13]. However, the exact causes of this variation across different populations are not fully understood. The risk of preterm birth has been demonstrated to vary by season, being marked by changes in physical activity, temporal spikes in various types of infection, or variation in climatological and environmental factors (e.g., temperature, air pollution, photoperiod) [12,13,14,15,16]. Social, cultural, and economic factors influencing sexual activity may also play a role, as well as times of economic and social uncertainty having a negative impact on pregnancy [8,17,18]. One such period of global uncertainty, which may have influenced preterm birth patterns, was the COVID-19 pandemic.

The COVID-19 pandemic caused by SARS-CoV-2 began in December 2019 and spread worldwide in 2020. The outbreak was declared a public health emergency, and governments took various measures to limit the health and environmental consequences of the pandemic. Restrictions included mitigation measures, lockdowns, mask mandates, quarantines, and the closure of educational institutions and public areas. These actions led to an increased focus on hygiene and social distancing, resulting in reduced microbial exposure. However, healthcare systems were profoundly affected, and the social and economic consequences were substantial. These extensive changes prompted researchers to investigate whether the pandemic had any observable effect on pregnancy outcomes, especially preterm birth.

Several studies conducted in Ireland, Germany, Denmark, the USA, Australia, and other countries found a reduction in preterm births during the COVID-19 lockdown period [19,20,21,22,23,24]. Other studies reported inconsistent evidence for a reduction in preterm birth linked to the COVID-19 pandemic or its lockdown measures [25,26,27]. A meta-analysis evaluating preterm birth and stillbirth during the COVID-19 lockdown in 26 countries demonstrated a reduction in preterm birth only in high-income countries [28]. Chmielewska et al. confirmed these findings while reporting an increase in stillbirths in low-income and middle-income countries [29]. Zeitlin et al. also reported similar findings, analyzing how preterm birth and stillbirth rates vary by socioeconomic status across European countries [30]. These inconsistent findings highlight the importance of region-specific approach investigations.

In Romania, the first positive COVID-19 case was detected in February 2020, and a nationwide state of emergency was declared starting in March 2020. Restrictions began to be gradually lifted in May 2020, yet a state of alert persisted until March 2022. The governmental mitigation measures included a mask requirement in public spaces, prohibition of movement outside of households with few exceptions, and the closure of all non-essential public institutions. To maximize the efficiency of the healthcare system, ambulatory facilities were closed, all non-urgent admissions and interventions were postponed, and healthcare personnel were redistributed to COVID-19 cases. Consequently, Romanian studies analyzing the effects of the COVID-19 pandemic on perinatal outcomes indicated an increase in stillbirths, while reports on the preterm birth rate were inconsistent [23,31,32,33,34].

We hypothesize that the COVID-19 pandemic, along with the associated lockdown measures, contributed to significant changes in the incidence of preterm birth. By analyzing the factors involved, this study aims to generate new insights that can support efforts to reduce the risk of prematurity. The objective of this study is to evaluate the impact of the COVID-19 pandemic and associated lockdown measures on the seasonality and prevalence of preterm birth in two regions of Romania, across three distinct time periods: before the pandemic, during the pandemic and lockdown, and after the pandemic.

## 2. Material and Methods

### 2.1. Study Setting

This population-based retrospective cohort study was conducted in two different counties of Romania, Bihor, and Sibiu. Bihor County is Romania’s 6th largest county, located in the Northwest, with a population of 600,000 people. Sibiu County has a population of about 400,000 people and is located in the center of the country, separated from the southern part of the country by the Carpathian Mountains. While Bihor has a continental-moderate climate, Sibiu is characterized by sharper differences between seasons and colder weather throughout the year [35]. Both counties have a single tertiary state maternity hospital [36,37].

This study was conducted and reported in accordance with the STROBE (Strengthening the Reporting of Observational Studies in Epidemiology) guidelines. A completed STROBE checklist is provided in the Supplementary Materials.

### 2.2. Data Sources and Study Population

**Figure 1:**
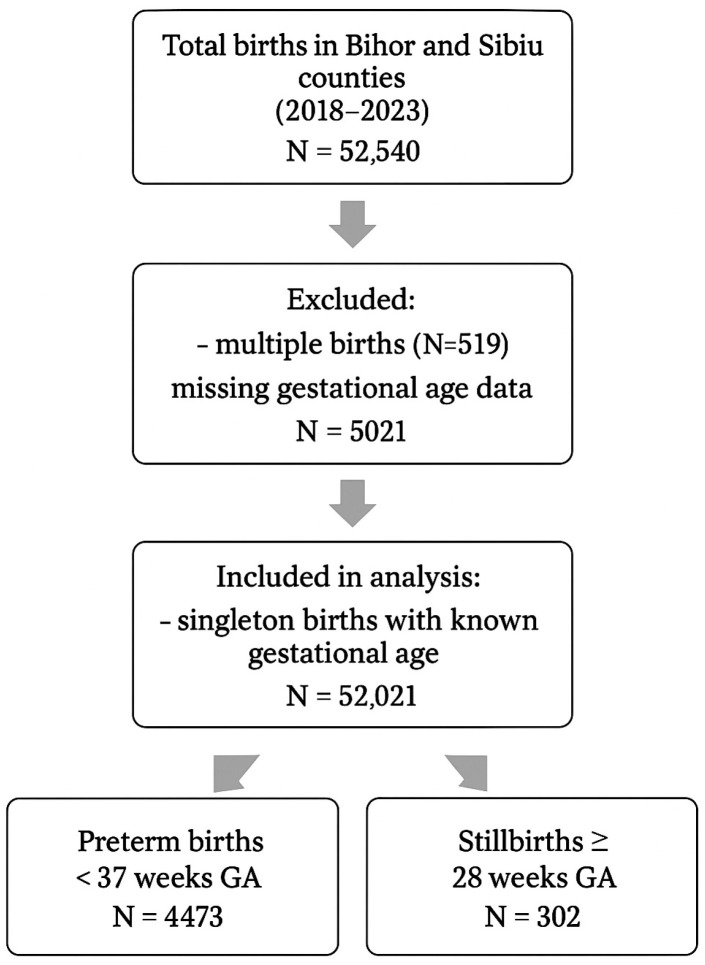
Study flowchart with inclusion and exclusion criteria.

We analyzed all singleton preterm births (*n* = 4473) and stillbirths (*n* = 302) in Bihor and Sibiu Counties over 6 years (2018–2023) (Figure 1). The incidence of prematurity and stillbirth during this period was determined and compared between the two counties. To detect associations between the COVID-19 pandemic and lockdown on prematurity and stillbirth rate, we compared the incidences between the pre-pandemic (2018, 2019, and January, February 2020), pandemic (March–December 2020, 2021, and January, February 2022), and post-pandemic periods (March–December 2022, 2023). To account for seasonal variability, six intervals during the analyzed periods were also compared: (1) pre-pandemic winter (January–February–December 2018, January–February–December 2019, January–February 2020) and summer periods (June–August 2018 and June–August 2019), (2) pandemic winter (December 2020, January–February–December 2021, January–February 2022) and summer periods (June–August 2020 and June–August 2021), and (3) post-pandemic winter (December 2022, January, February 2023) and summer periods (June 2022–August 2022 and June 2023–August 2023). Additionally, a comparison was made between the mentioned periods and the strict lockdown period (March–May 2022).

### 2.3. Definitions and Measurements

The preterm birth rate was defined as the number of live-born infants with a gestational age between 24+0/7 and 36+6/7 weeks per 1000 live births.

The Romanian legislation classifies stillbirth as fetal death from 28 weeks of pregnancy onward. The stillbirth rate refers, therefore, to the number of infants born in a hospital without signs of life, with a gestational age of 28+0/7 weeks or more, per 1000 births [38]. Gestational age was determined using the most accurate estimates from obstetric history, obstetric examinations, and the first prenatal ultrasound.

Preterm infants were categorized into moderate and late preterm (32+0/7 to 36+6/7 weeks of gestation), very preterm (<32+0/7 weeks of gestation), and extremely preterm infants (<28+0/7 weeks of gestation). Analysis was performed based on these gestational age groups.

### 2.4. Statistical Analysis

Incidence rates were used for the descriptive statistics. For the three periods examined (pre-pandemic, pandemic, and post-pandemic), the monthly incidence rates were totaled for each county. The Kruskal–Wallis test, a non-parametric statistical test, was used for comparing three or more groups. The Spearman coefficient was calculated to determine the correlation between variables. The significance level for all the statistical tests was established at a *p*-value of 0.05. The statistical analysis was conducted using IBM SPSS Statistics 23 software (IBM, Armonk, New York, NY, USA) and Microsoft Excel (Seattle, Washington, DC, USA).

### 2.5. Ethical Approval

This study received approval from the ethical committee of Clinical County Hospital Bihor and Clinical County Hospital Sibiu, Romania.

## 3. Results

### 3.1. Birth Rate

**Figure 2:**
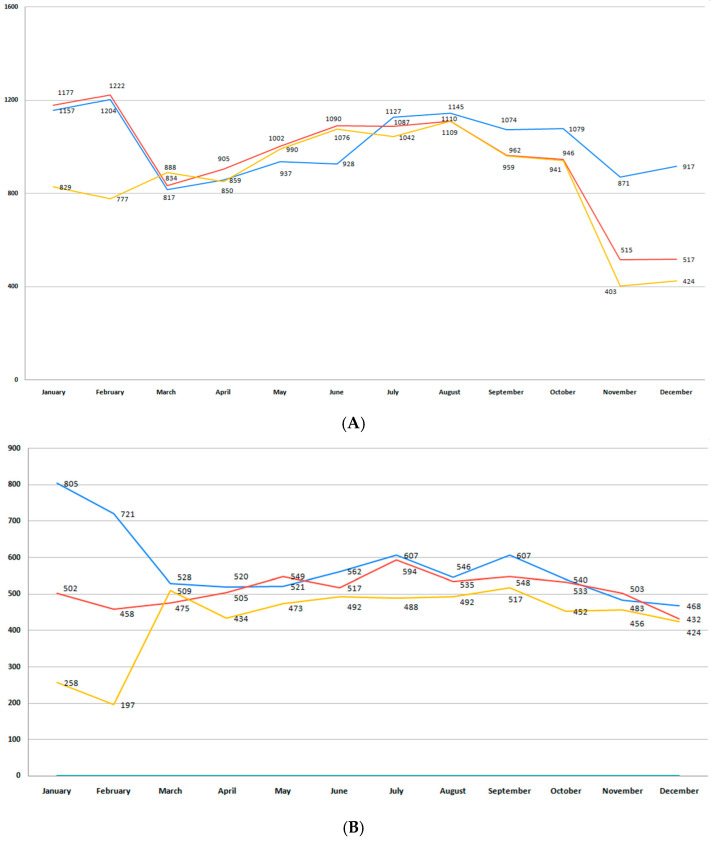
(**A**) Monthly number of births during the studied period for Bihor County. (**B**) Monthly number of births during the studied period for Sibiu County. (blue—number of births in the pre-pandemic period; red—number of births in the pandemic period; orange—number of births in the post-pandemic period).

This study included 52,021 births, 33,770 (64.9%) registered in Bihor County and 18,251 in Sibiu County. The total number of births per month during the studied period in each region is presented in Figure 2A,B. Bihor County had a higher number of births than Sibiu, with minor fluctuations in the last 6 years (*p* = 0.11) (Figure 2A). In Sibiu County, a higher number of births was registered during the pre-pandemic years, when compared to the post-pandemic period (*p* = 0.001) (Figure 2B).

These differences were not statistically significant when data from both counties were analyzed together (*p* = 0.92).

### 3.2. Incidence of Premature Birth

**Figure 3:**
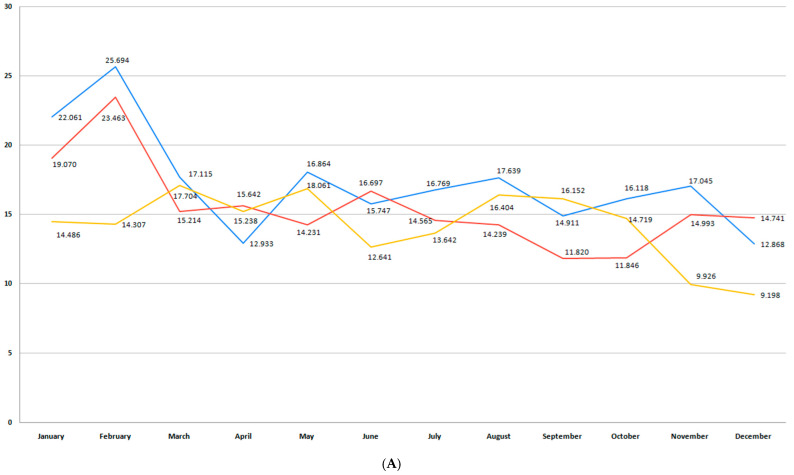

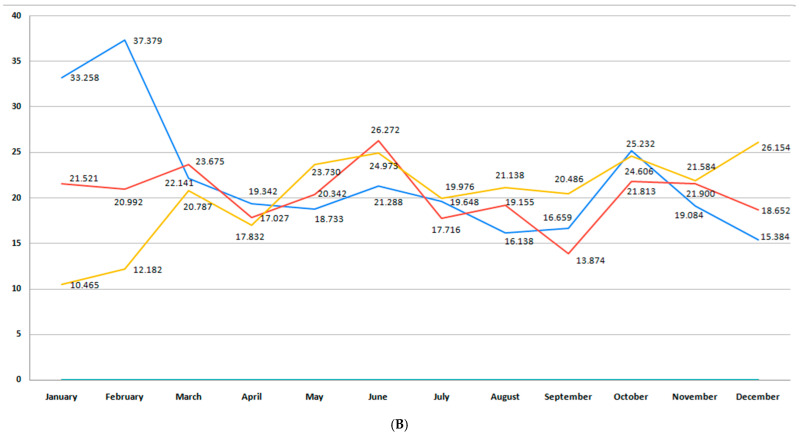
(**A**) Monthly number of preterm births during the studied period for Bihor County. (**B**) Monthly number of preterm births during the studied period for Sibiu County. (blue—number of births in the pre-pandemic period; red—number of births in the pandemic period; orange—number of births in the post-pandemic period).

In our study, the prematurity incidence varied between 8 and 10% during the 6-year period, depending on the region. Of 33,770 births registered in Bihor County, 2574 were premature births (7.62%). The total preterm birth rate in Sibiu County during the studied period was 10.4%. Aligned with the global trend, a continuous decline in preterm birth rate could be observed beginning with 2018 (Figure 3A,B). The highest rate of preterm births was recorded in both counties during the pre-pandemic period, particularly during the first trimester of the year. This trend also continued in the pandemic years in Bihor, while the preterm birth rate dropped in Sibiu County. The COVID-19 pandemic led to a reduction in preterm birth in the following years in both regions, as the orange trend line shows. However, these differences were not statistically significant (*p* = 0.13).

**Figure 4:**
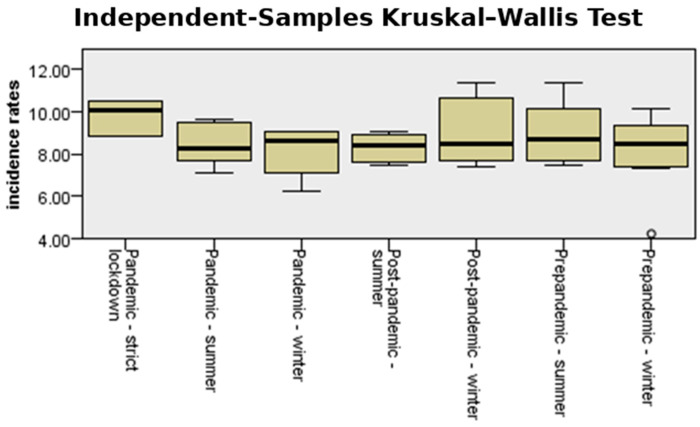
Independent-samples Kruskal–Wallis test for the variable “total incidence rate” in the studied cohort.

Figure 4 shows the incidence and interquartile range (IQR) of preterm birth for both counties in the analyzed periods, comparing pandemic-strict lockdown with pre-pandemic and post-pandemic seasonality of preterm birth. The median incidence of preterm birth in strict lockdown was much higher (10%) than that corresponding to the summer and winter periods, both in the pre-pandemic, pandemic, and post-pandemic periods. During the periods outside the lockdown, the median values varied between 8.25% and 8.68%.

**Figure 5:**
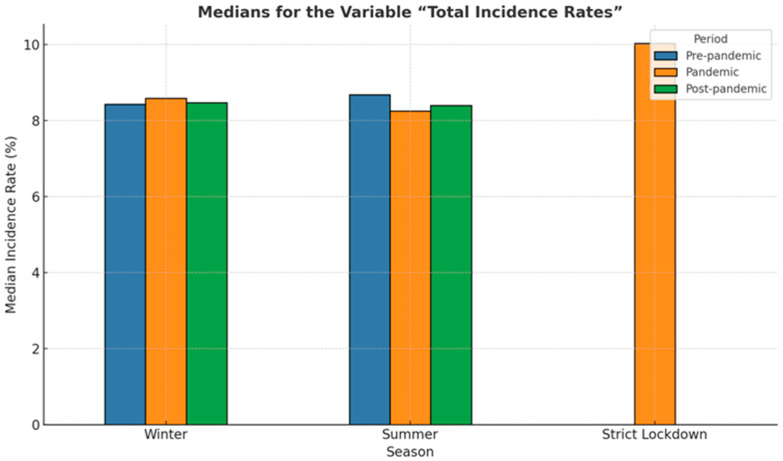
Bar plot illustrating medians of the premature birth incidence rate across three periods: pre-pandemic (blue), pandemic (orange), post-pandemic (green), and during strict lockdown.

Median rates of preterm birth during winter and summer seasons and the strict lockdown period are presented in Figure 5. The lowest median of prematurity incidence, 8.25%, was recorded during the pandemic summer period, while the highest (8.68%) was also recorded during summer but before the COVID-19 pandemic. Overall, in our cohort, medians in the winter periods were slightly lower than in the summer periods, regardless of whether they were in the pre-pandemic, pandemic, or post-pandemic periods. However, these differences were not statistically significant (*p* = 0.65).

### 3.3. Moderate and Late Preterm Birth (32+0/7–36+6/7 Weeks GA)

**Figure 6:**
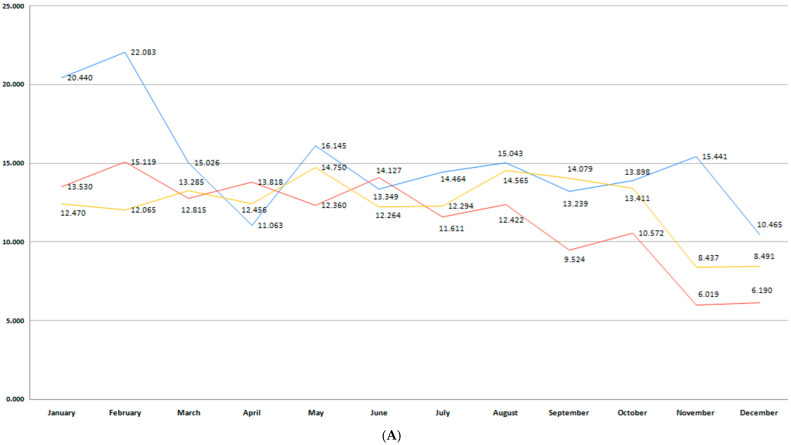

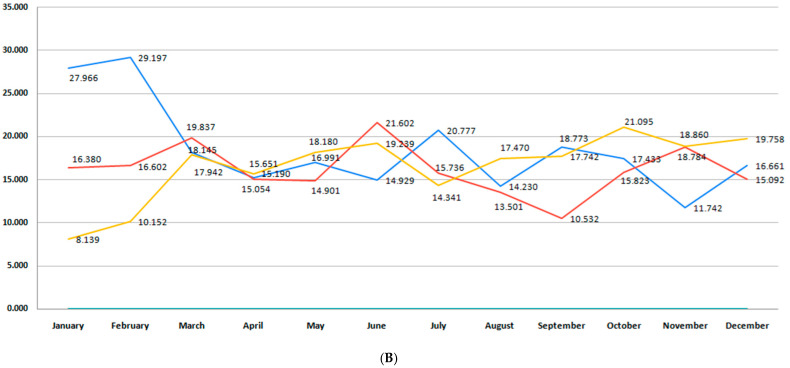
(**A**) Monthly number of moderate to late preterm births during the studied period for Bihor County. (**B**) Monthly number of moderate to late preterm births during the studied period for Sibiu County (blue—number of births in the pre-pandemic period; red—number of births in the pandemic period; orange—number of births in the post-pandemic period).

Both regions registered a drop in the number of moderate to late preterm births beginning in 2018. The blue bar in Figure 6A,B shows a peak for both counties during the first months of 2018, 2019, and 2020, with a notable decrease in moderate and late preterm infants in the following years. In the Bihor region, this category of premature infants experienced a continuously declining rate during the pandemic period, as the red trend line illustrates (Figure 6A).

No difference in the rate of moderate to late preterm birth could be observed during the studied period (*p* = 0.17).

**Figure 7:**
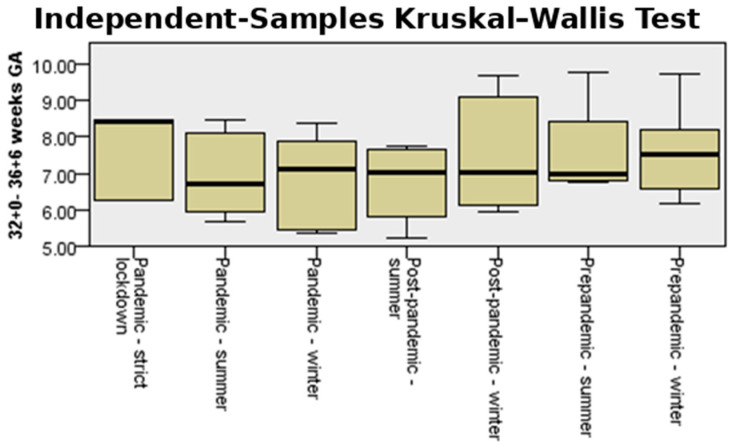
Independent-samples Kruskal–Wallis test for the variable “moderate to late preterm birth”.

Figure 7 illustrates the incidence of prematurity for the moderate to late preterm group summed up for the entire cohort. In this category, the highest incidence was also reached during the pandemic strict lockdown period.

**Figure 8:**
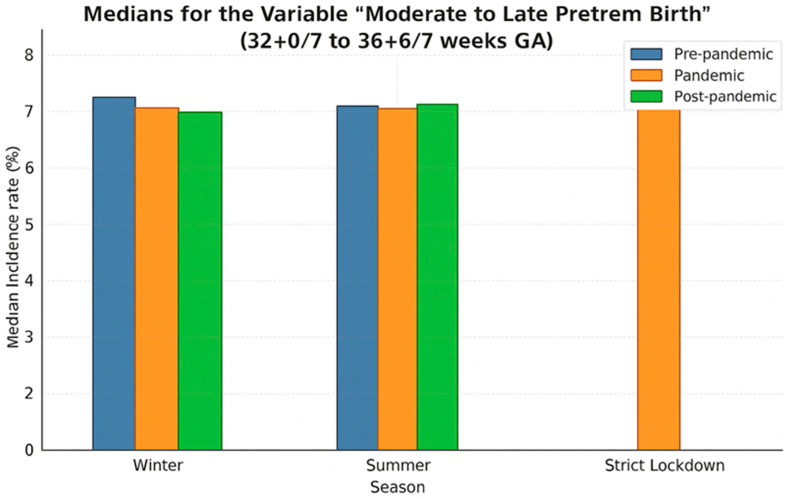
Bar plot illustrating medians for the variable “moderate to late preterm birth” across three periods: pre-pandemic (blue), pandemic (orange), post-pandemic (green), and during strict lockdown.

As Figure 8 shows, the median incidence of preterm birth during the summer in the pre-pandemic and pandemic years was smaller than that in winter (7.00 vs. 7.52, respectively, 6.73 vs. 7.12; *p* = 0.78). However, this trend reversed after the COVID-19 pandemic.

The lowest median incidence of moderate to late preterm birth corresponded to the summer period of the pandemic. During the strict lockdown period, the median incidence was 8.42% higher compared to the periods outside the lockdown (Figure 8).

### 3.4. Very Preterm Birth (28 0/7–31 6/7 Weeks GA)

**Figure 9:**
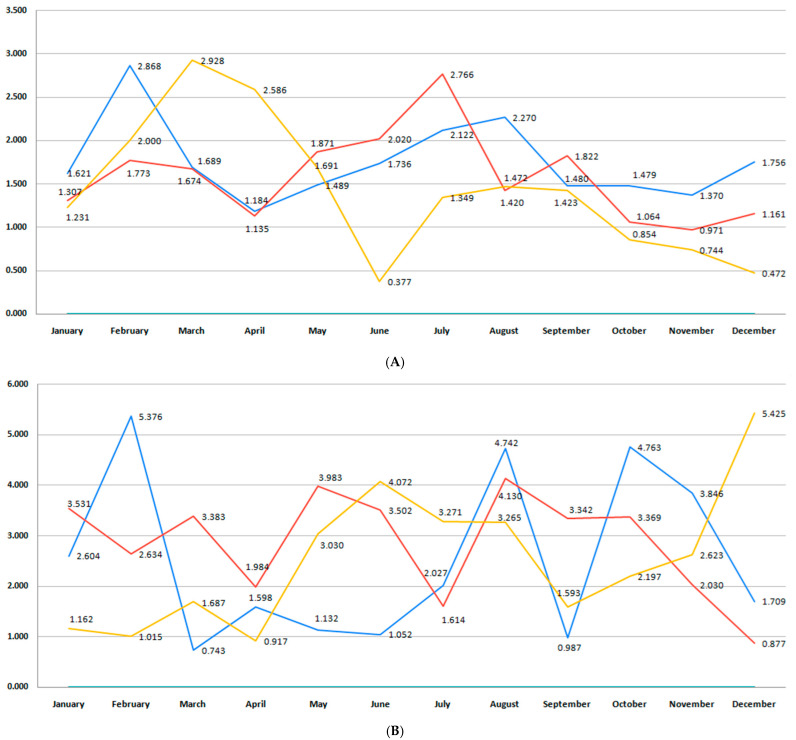
(**A**) Monthly comparison of very preterm birth rate during the studied period for Bihor County. (**B**) Monthly comparison of moderate to late preterm birth rate during the studied period for Sibiu County (blue—pre-pandemic period; red—pandemic period; orange—post-pandemic period).

The number of very preterm births declined beginning with 2018 in both regions, and this trend continued with some variations during the pandemic years, as the blue and red lines show in Figure 9A,B. However, some differences were noted when analyzing Bihor and Sibiu counties separately in the years after the COVID-19 pandemic. The Bihor region was characterized by a peak of very preterm births in the first half of 2022 and 2023, with a decline during autumn and winter (Figure 9A). In Sibiu, the graph reveals marked monthly variability and multiple peaks across all three timeframes. The pre-pandemic period reveals striking peaks in February (5.37%) and September (4.74%), followed by another in October (4.76%), indicating a potential pattern of early- and late-year vulnerability. The post-pandemic period follows a similar trajectory, with elevated rates in May (3.98%), August (4.13%), and October (3.36%). In contrast, the pandemic period shows slightly lower but still erratic peaks, with notably high rates in June (4.07%) and December (5.42%), which might reflect inconsistent care access, delayed presentation, or differences in risk profiles during the lockdown period. There was no difference in very preterm birth incidence between the pre-pandemic, pandemic, and post-pandemic period when data for both counties were analyzed together (*p* = 0.94).

**Figure 10:**
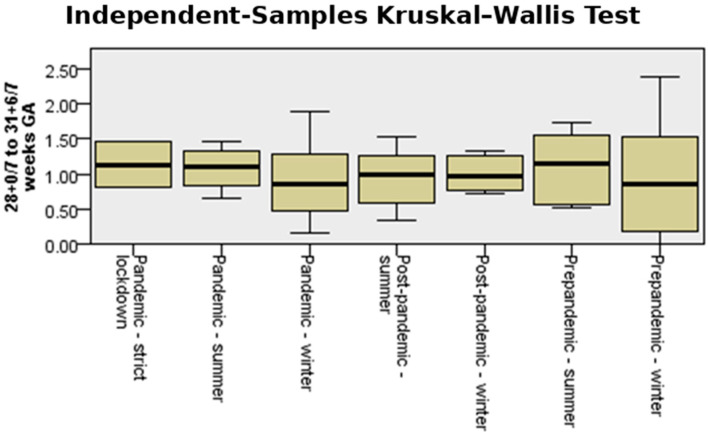
Independent-samples Kruskal–Wallis test for the variable “very preterm birth”.

Figure 10 presents the incidence and IQR for very preterm births for both counties in the analyzed periods, comparing the pandemic-strict lockdown with pre-pandemic and post-pandemic seasonality of preterm birth. The median rates during winter were slightly lower than during summer, regardless of whether it was pre-pandemic, pandemic, or post-pandemic. However, the observed differences were not statistically significant (*p* = 0.97).

**Figure 11:**
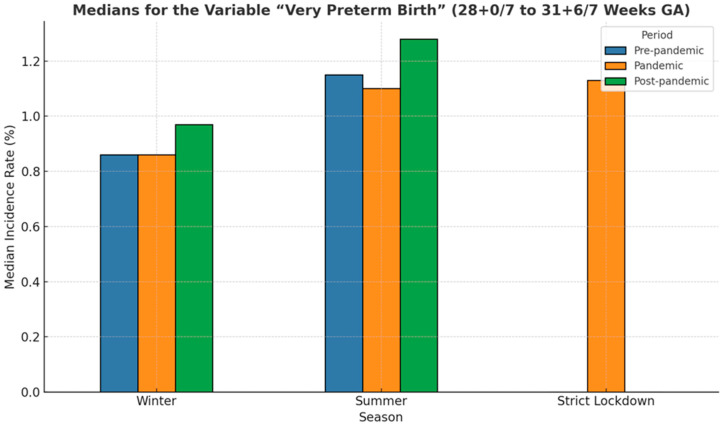
Bar plot illustrating medians for the variable “very preterm birth” across three periods: pre-pandemic (blue), pandemic (orange), post-pandemic (green), and during strict lockdown.

During strict lockdown, the median incidence was similar to the one recorded usually during the summer season for this category of prematurity, irrespective of the period, before, during, or after the pandemic (around 1.1) (Figure 11).

### 3.5. Extremely Preterm Birth (<28 Weeks GA)

**Figure 12:**
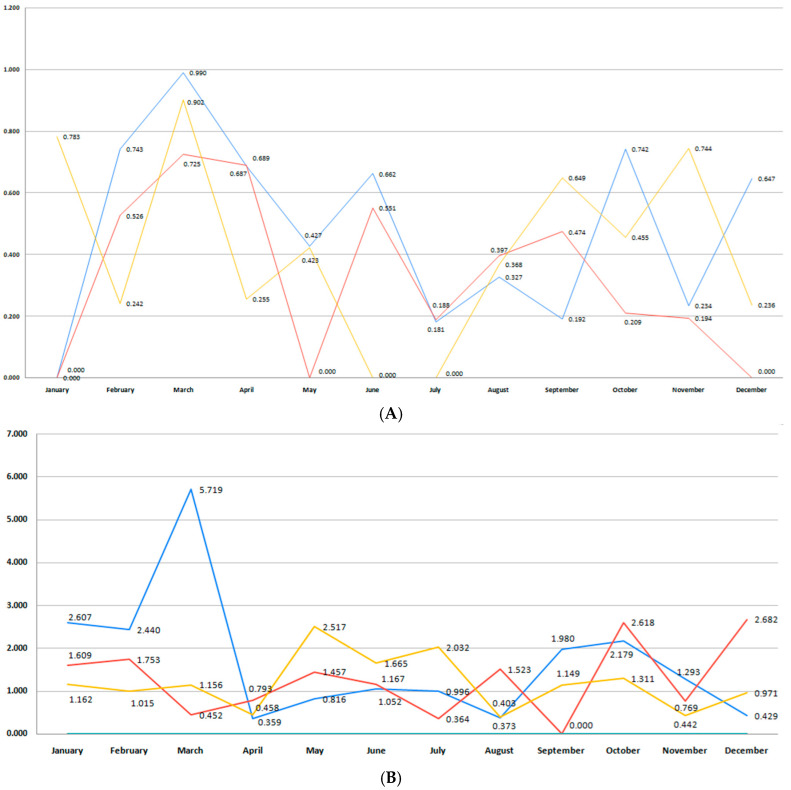
(**A**) Monthly comparison of extremely preterm birth rate during the studied period for Bihor County. (**B**) Monthly comparison of extremely preterm birth rate during the studied period for Sibiu County. (blue—pre-pandemic period; red—pandemic period; orange—post-pandemic period).

Differences in extremely preterm birth rates were observed between the two regions during the 6-year period. The Bihor region is characterized by a higher total number of infants born at less than 28 weeks than Sibiu. For Sibiu, a significant outlier is seen in March of the pre-pandemic period, with the highest recorded rate of 5.71%, far exceeding other values across all three timelines. Beginning with the first trimester of 2018 and in 2019, we observed a decrease in the rate of births with a gestational age of less than 28 weeks (Figure 12B). In this county, a reduced incidence of extremely preterm birth persisted after the pre-pandemic years, with some minor variations (*p* = 0.94). In the Bihor region, we observed fluctuations around an incidence rate of 0.6%. In the pre-pandemic period, the highest incidence occurred in March (0.99%) and June (0.86%), with relatively consistent moderate rates in early- and mid-year months. During the pandemic period, notable spikes appeared in January (0.78%), March (0.90%), and October (0.74%), while several months recorded zero incidence, likely due to the smaller number of events (Figure 12A).

Analyzing all data together, there was no difference between the extremely preterm birth rate during the pandemic years and the rest of the analyzed period (*p* = 0.72).

**Figure 13:**
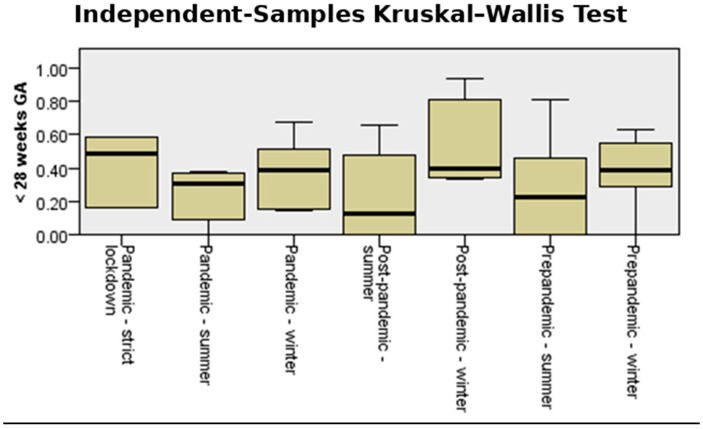
Independent-samples Kruskal–Wallis test for the variable “extremely preterm birth”.

The number of infants born at less than 28 weeks during the studied period fluctuated slightly when both counties were analyzed together. However, the highest median was also recorded during the strict lockdown period (Figure 13).

**Figure 14:**
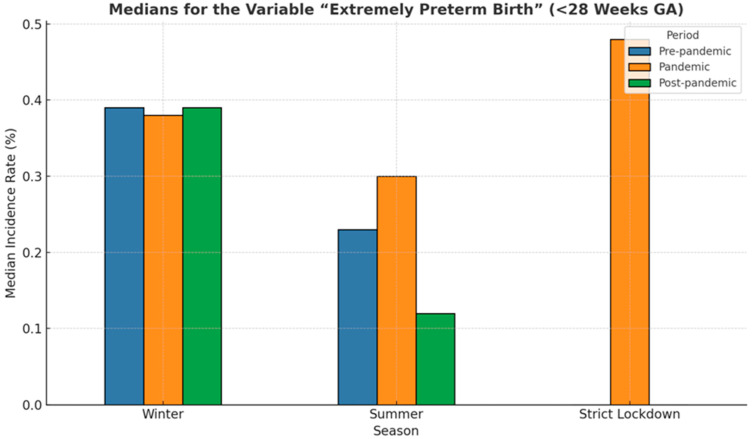
Bar plot illustrating medians for the variable “extremely preterm birth” across three periods: pre-pandemic (blue), pandemic (orange), post-pandemic (green), and during strict lockdown.

The median incidence during winter and summer registered in the strict lockdown period varied during the analyzed 6-year period between 0.39% in the winter before COVID-19 to 0.12%, the value recorded in the summer that followed the global pandemic (Figure 14). Although the differences are not statistically significant (*p* = 0.38), the median rates for the summer season were lower than those corresponding to the winter period, regardless of whether they were in the pre-pandemic, pandemic, or post-pandemic years.

### 3.6. Incidence of Stillbirth

**Figure 15:**
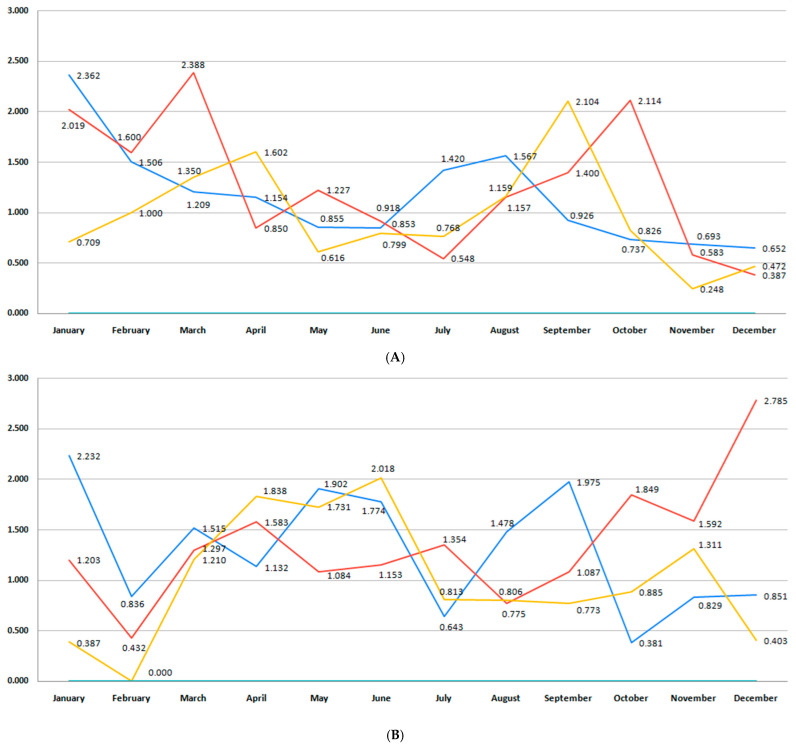
(**A**). Monthly stillbirth rate during the studied period for Bihor County. (**B**) Monthly stillbirth rate during the studied period for Sibiu County. (blue—pre-pandemic period;red—pandemic period; orange post-pandemic period).

The stillbirth rate did not reach statistically significant differences during the 6-year analysis (*p* = 0.52). However, the blue trend line for the Bihor region in Figure 15A shows a decreasing trend in 2018–2019, a trend that was reversed in the next 2 years. The pandemic period (red) is characterized by two of the highest stillbirth rates, occurring during the winter of the studied two-year period (2022–2023) (2.38%) and the autumn season (2.11%). By contrast, the post-pandemic period (orange) exhibits lower rates in most months, although there are still intermittent peaks (e.g., April and September 2020–2021). Figure 15B presents monthly stillbirth rates in Sibiu County across the three distinct periods. The graph shows a high degree of month-to-month variability, particularly in the pre-pandemic and post-pandemic periods, with several pronounced peaks. The highest stillbirth rate was recorded in December of the pandemic period (2.78%). By contrast, several months during the post-pandemic period had notably lower or zero values, suggesting potential underreporting or temporary fluctuations possibly related to changes in maternal behavior or improved antenatal care.

**Figure 16:**
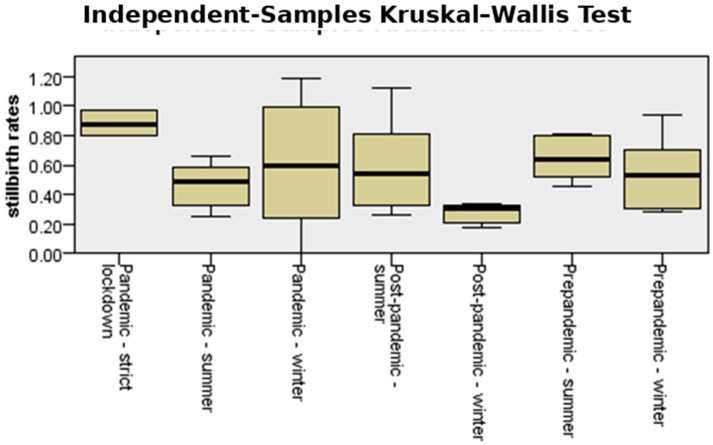
Independent-samples Kruskal–Wallis test for the variable “stillbirth”.

When seasonality of stillbirth was analyzed, a significant difference in stillbirth rate could be observed during the strict lockdown period compared to other periods before and after the pandemic (*p* = 0.05), as shown in Figure 16.

**Figure 17:**
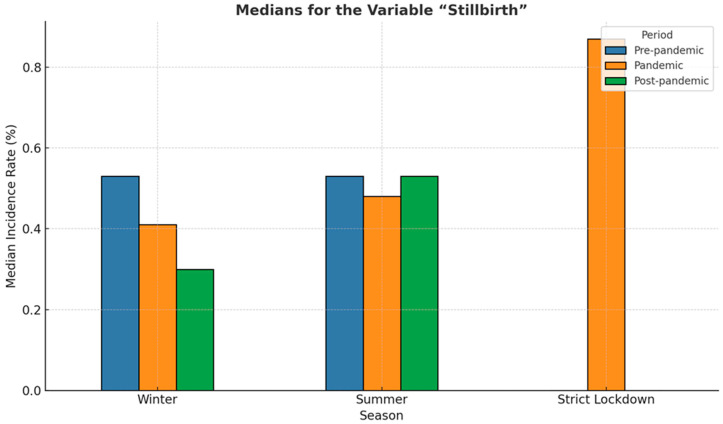
Bar plot illustrating medians for the variable “stillbirth” across three periods: pre-pandemic (blue), pandemic (orange), post-pandemic (green), and during strict lockdown.

Figure 17 presents the median incidence of stillbirth analyzed for the winter and summer seasons and the strict lockdown period. The values recorded during the strict lockdown period were almost double compared to the other periods (*p* = 0.05).

Statistical indicators, including mean and standard deviation for the variable stillbirth, are presented in Table 1. The analysis of stillbirth incidence across different periods and seasons revealed notable variation in both central tendency and dispersion. During the strict lockdown period, the mean stillbirth rate was the highest at 0.88%, with a relatively low standard deviation (SD = 0.08), indicating a consistently elevated rate across observations. In contrast, the post-pandemic winter period recorded the lowest mean stillbirth rate at 0.28% (SD = 0.06), with minimal variability.

The pandemic-winter period showed a mean similar to the pre-pandemic winter (0.52% vs. 0.53%) but was characterized by markedly higher variability (SD = 0.43), suggesting unstable rates during this interval. Notably, the post-pandemic summer period had the widest range (0.26 to 1.11) and a relatively high mean (0.59%) and standard deviation (SD = 0.32), indicating significant fluctuations in stillbirth incidence during this season.

## 4. Discussion

Seasonality is a big problem in allocating resources. The infrastructure (NICU size, ventilators, infusion pumps) is a capital allocation, but the allocation of human resources (nurses, doctors, support staff) is a more complex and expensive question. The need for vacation results in some countries in the temporary closure of available NICU beds, which does not necessarily coincide with the fluctuations of preterm birth. The resulting quest to find a bed for preterm in utero is one thing, but to have the preterm infant transported immediately after birth affects the outcome in a negative manner [39,40,41].

We took the events of the pandemic as an opportunity to analyze the seasonality in dependence of lockdown, social distancing, hand hygiene, and face masks. In addition, we compared the seasonality of the year in two distinct counties where the extremes of summer and winter are somewhat different in the context of Eastern European climate [35]. Seasonal variation in preterm birth has been reported in multiple epidemiological studies and is thought to reflect fluctuations in environmental exposures. In this context, we considered it relevant to evaluate whether the patterns observed during the COVID-19 pandemic aligned with or diverged from established seasonal trends. Incorporating seasonal stratification into our analysis allowed us to investigate whether pandemic-related lifestyle and healthcare disruptions amplified or mitigated these seasonal effects on preterm birth and stillbirth [7,12,13,14]. The strict lockdown period (March–May 2020) was examined as a distinct time frame in this study due to its unique public health context. Unlike the broader seasonal comparisons (summer and winter), this short interval was marked by exceptional systemic disruption, including nationwide restrictions on movement, suspension of non-essential medical services, and reorganization of hospital infrastructure to address the COVID-19 crisis. These unprecedented measures had the potential to affect perinatal outcomes independently of seasonal variation. By isolating the lockdown period as a comparator, we aimed to assess whether such acute changes in healthcare access and daily life were associated with measurable differences in preterm birth and stillbirth rates. This approach allowed us to differentiate between gradual, seasonally driven changes and those more directly linked to the abrupt societal and institutional shifts caused by pandemic containment efforts.

In this cohort involving more than 50,000 births across two different counties in Romania, no differences were found in the incidences of the main adverse pregnancy outcomes—prematurity and stillbirth—when comparing the COVID-19 period with the time period in 2018 and 2019 combined (pre-pandemic period) or 2022 and 2023 combined (post-pandemic period). However, when assessing seasonality, a positive correlation was found between stillbirth incidence and the strict lockdown period (*p* = 0.05). The incidence of prematurity showed no significant changes during stringent mitigation measures. Similar results were observed when preterm subtypes were analyzed separately.

In our study, birth rate did not undergo significant changes over the 6-year period analyzed. Lower birth rates were observed during the two-year pandemic period, especially 9 months after the pandemic onset, corresponding to the months of November and December 2020, especially in Bihor County (Figure 2A). However, when data from both regions were pooled, there were no differences in birth rate between the pre-pandemic, pandemic, and post-pandemic period (*p* = 0.92). According to national data from the Institute of Public Health, 2020 marked Romania’s lowest birth rate in three decades [42]. In both counties, birth rates dropped during 2020 and 2021, considered as the pandemic years [43,44]. International studies have also reported a decrease in birth rates during the COVID-19 pandemic [45,46]. Possible causes leading to a drop in the total number of births are related to the economic uncertainty, changes in fertility, social fears, social distancing, or the population-wide vaccination campaign. However, the short-term decrease in birth incidence is associated with a rebound in the number of births following the pandemic, as studies report [47,48,49].

The incidence of prematurity in our cohort varied between 8% and 10% during the analyzed 6-year period. Preterm birth rates remained statistically unchanged during the COVID-19 pandemic compared with the periods before and after the pandemic (*p* = 0.13). However, this *p*-value may suggest a potential trend that could warrant further investigation. Given the retrospective nature of the study, we interpret this result with caution, recognizing the need for larger studies to explore whether this trend reflects a meaningful difference. The incidence of preterm birth during COVID-19 has been analyzed by different studies, reporting inconsistent findings. Meta-analyses by Vaccaro et al. [50] and Yang et al. [51] showed no differences in pooled ORs. Chmielewska et al. [29] and a meta-analysis with data from 26 countries [28] confirmed a reduced incidence of preterm birth only in HIC. In contrast, our findings align more closely with the results from low- and middle-income countries, where such reductions were not observed. One possible explanation is that in the Romanian context, any potential protective factors during lockdown—such as reduced physical activity or infectious exposures—may have been outweighed by persistent risk factors, including prolonged psychosocial stress, limitations in healthcare system capacity, and reduced access to timely, high-quality antenatal care. These factors may have prevented the emergence of any measurable decline in preterm birth incidence, emphasizing the critical role of health system resilience during public health emergencies. Within our cohort, there was no observed seasonal variation in preterm birth rates when comparing summer and winter incidences across pre-pandemic, pandemic, and post-pandemic years, including the lockdown period. Existing literature research has demonstrated a seasonal pattern of preterm birth [7,52]. This pattern differs between countries and may be impacted by climatic factors, such as ambient temperature, length of daylight, atmospheric pressure, or humidity [8,52,53]. Moreover, social customs and economic and cultural factors are also involved [54]. The inclusion of two distinct geographic regions—Bihor, characterized by a continental-moderate climate, and Sibiu, located in a mountainous area with sharper seasonal variations—allowed us to explore whether environmental and climatic differences might contribute to variations in preterm birth and stillbirth rates. Regions such as Sibiu are subject to lower average temperatures and greater seasonal fluctuation, which may affect maternal behavior, healthcare accessibility, and exposure to respiratory or other infections. Although no significant regional differences in perinatal outcomes were observed in our data, the potential influence of climate on seasonal trends remains an area worth exploring in future studies with more environmental data.

The absence of a consistent seasonal pattern in preterm birth rates in our study aligns with findings from several other temperate-climate countries, for example, the United Kingdom [53], suggesting that temperature and daylight variation alone may not be sufficient to drive changes in birth timing. In HICs, such as the USA and Japan, winter and summer peaks in incidence have been reported [16,44,48]. In a London-based cohort study [53], a peak was only observed in winter, with colder temperatures being associated with increases in the risk of preterm birth, as demonstrated by a European meta-analysis of birth cohorts [55]. When moderate to late preterm births, very preterm births, and extremely preterm births were analyzed separately, no differences in incidence were found between the pre-pandemic, pandemic, and post-pandemic periods. Similarly, no seasonal associations were found.

As compared to other studies, no reduction in extremely preterm birth during the COVID-19 pandemic (*p* = 0.72) or during the lockdown was observed. Seasonal variability in the incidence of extreme prematurity was not found during the 6-year period (*p* = 0.38); however, median incidences of prematurity tended to be lower during winter, as compared to summer (Figure 14). Hviid et al. [56] assessed the seasonality of extremely preterm birth in a Danish cohort of more than 1 million pregnancies, and reported an association of only 2.8% of all extremely preterm births with seasonality. In this study, the lowest rate of extremely preterm birth was also recorded during winter.

Another important adverse pregnancy outcome that is closely linked to preterm birth is stillbirth. We found an association between the stillbirth rate and the COVID-19 lockdown. While no consistent seasonal trend was observed across the years (*p* = 0.52), the data suggest that pandemic-related disruptions, especially during the strict lockdown, were associated with an increase in stillbirth incidence and less variability compared to other periods. These findings highlight the potential impact of constrained healthcare access and pandemic stressors on perinatal outcomes. In Bihor, the highest number of stillbirths during the analyzed period was registered during the pandemic period (Figure 15A). For Sibiu, a peak was recorded about nine months after the onset of the COVID-19 pandemic (Figure 15B). These trends suggest that while seasonal variation may not be strongly deterministic, stillbirth rates in Sibiu may have been influenced by healthcare access, reporting patterns, and population behavior across different stages of the pandemic. Overall, the data demonstrate substantial inter-month variation, reinforcing the importance of monitoring regional differences and ensuring continuity of maternal care during and after public health emergencies. Data released by the National Public Health Institute shows an increase in the stillbirth incidence for the majority of the counties in Romania during 2020 [57]. However, this trend reversed during 2021 for the majority of the counties in Romania [58,59]. International studies reported conflicting data regarding the stillbirth rate during the pandemic. While some found no change in stillbirths, others concluded that changes in the stillbirth rate may depend on a country’s socioeconomic status, with low- and middle-income countries associated with a higher risk of an increase in stillbirths [60,61,62,63]. A study analyzing changes in preterm birth and stillbirth rates in 21 European countries reported an increase in stillbirth incidence independent of the socioeconomic status of the country [30]. Several other international studies also analyzed stillbirth incidence during lockdown, and findings from both LMIC and HIC are similar to ours, reporting an increase in stillbirth rate during the lockdown [64,65].

An increase in stillbirths may have resulted as a direct consequence of maternal infection with SARS-CoV-2 or as an indirect outcome of pandemic-related disruptions in healthcare delivery. While our data does not allow us to distinguish between mothers who tested positive and negative for COVID-19 in the stillborn infants’ cohort, any direct link between COVID-19 infection and stillbirth would likely have led to an increased rate of stillbirths throughout the pandemic period. Instead, the observed rise was temporally concentrated during the strict lockdown phase. Alternatively, the increase in stillbirths may stem from indirect factors associated with lockdown measures. Examples are reduced access to timely, quality antenatal care due to social distancing measures, reallocation of personnel for managing the healthcare crisis, and the closing of private healthcare facilities [66,67,68]. Additionally, pregnant women might hesitate to seek hospital care when needed (e.g., when experiencing decreased fetal movements) due to fears of contracting the COVID-19 infection.

The rise in stillbirths within this population highlights the importance of closely monitoring the mother–fetus dyad during challenging public health emergencies, such as the COVID-19 pandemic. Additionally, it is crucial to monitor changes in care-seeking behavior and the quality of labor and delivery services in public health facilities, as these factors may contribute to adverse outcomes.

Romania’s healthcare context may have uniquely influenced the observed outcomes. As a middle-income country with regional disparities in healthcare accessibility, Romania faced particular challenges during the pandemic [69,70]. Public hospitals are the primary providers of maternity care, and private sector services were significantly reduced during lockdown. Moreover, the suspension of outpatient services, reallocation of staff to COVID-19 units, and delays in non-urgent care further strained perinatal health services. In rural or underserved areas, pregnant women may have encountered additional barriers such as transport limitations, fewer healthcare providers, and fear of infection. These systemic vulnerabilities likely contributed to the pandemic’s differential impact on perinatal outcomes in Romania and may limit the generalizability of our findings to settings with more robust healthcare systems. Nevertheless, the combination of seasonal analysis and lockdown stratification in this context offers globally relevant insights into how healthcare infrastructure and emergency policy responses can interact to shape maternal and neonatal health.

Clinically, these findings underscore the importance of regionally tailored perinatal surveillance, especially during public health emergencies. Understanding how environmental, healthcare system, and social factors interact during crises like the COVID-19 pandemic is essential for optimizing antenatal care delivery. These insights can inform the development of responsive, climate- and context-sensitive maternal health policies and help ensure that perinatal surveillance systems remain robust and adaptive during future disruptions.

### Strengths and Limitations

The strength of our study lies in our examination of perinatal events together (preterm birth and stillbirths) within a large cohort of patients from two centers in Romania over a six-year period. We compared the outcomes of the pandemic period with two historically identical periods: the pre-pandemic two years and the post-pandemic two years. Moreover, to better understand the effects of COVID-19 and its associated lockdown measures, we compared the seasonality of preterm birth and stillbirth during summer and winter across the pre-pandemic, pandemic, and post-pandemic periods with the strict lockdown period. Another strength of this study is the completeness of the dataset, with no missing values for key variables such as gestational age and birth outcomes, thereby enhancing the reliability of the analyses and eliminating potential bias due to data exclusion or imputation.

The following limitations are acknowledged in this study: First, the data registration system did not allow us to differentiate between mothers infected with COVID-19 and those who were uninfected. This limitation hindered our ability to explore the specific impact of COVID-19 infection on preterm birth or stillbirth. Second, due to the retrospective design of the study, we were unable to account for all relevant clinical and sociodemographic factors associated with preterm birth, including detailed data on antenatal care utilization and maternal comorbidities. Important factors such as maternal vaccination status, access to antenatal care services, and socioeconomic status were not captured in the available dataset. These variables may have influenced both exposure and outcome, particularly during the COVID-19 pandemic. Furthermore, although appropriate non-parametric methods (such as the Kruskal–Wallis test) were used for group comparisons, the absence of post hoc pairwise testing with Bonferroni correction represents a limitation. This was primarily due to structural constraints in the dataset, which did not support the assumptions required for reliable subgroup analysis. Similarly, while advanced time series methods such as Joinpoint Regression could have enhanced the assessment of temporal trends, these techniques could not be applied due to the lack of sufficiently granular, time-stamped data. These approaches will be considered in future studies when more detailed datasets become available. Moreover, the use of retrospective registry data raises the possibility of reporting bias, especially during periods of healthcare system strain, where underreporting or inconsistent data entry may have occurred. These factors may limit the accuracy and generalizability of our findings and should be addressed in future prospective studies with more detailed and standardized data collection.

Lastly, while our findings suggest a potential association between socio-environmental changes during the COVID-19 pandemic and limited access to prenatal care—particularly in a low- and middle-income setting—this study was not designed to assess causality. Future prospective studies are needed to explore these associations more comprehensively.

## 5. Conclusions

Lockdown conditions are associated with significant changes in perinatal outcomes independent of the effect of the COVID-19 disease. Our study indicated that pregnant women exposed to a lockdown were more likely to suffer stillbirth, while rates of preterm birth remained unaffected by the mitigation measures or by altered maternal health-seeking behavior. As information accumulates on the effects of the COVID-19 pandemic lockdown globally, increasingly heterogeneous perinatal outcomes across countries become evident. Independent of the impact of the COVID-19 disease, pandemic-related restrictions were linked to significant changes in pregnancy outcomes.

More research is required to determine whether the observed changes in stillbirth rates are related to access to healthcare services, maternal health conditions and behaviors, or broader social and environmental factors. These findings highlight the critical need to ensure continuity and accessibility of antenatal care services within the healthcare system, particularly during public health crises, to help reduce the risk of adverse perinatal outcomes such as preterm birth and stillbirth.

## Figures and Tables

**Table 1:** Statistical indicators for the variable “stillbirth”.

Stillbirths	Minimum	Maximum	Mean	Std. Deviation
Strict lockdown	0.803	0.972	0.88237	0.085405
Pre-pandemicwinter	0.287	0.936	0.53387	0.236241
Pandemicwinter	0.000	1.119	0.52128	0.427681
Post-pandemicwinter	0.179	0.333	0.27865	0.068955
Pre-pandemic summer	0.287	0.812	0.56765	0.210876
Pandemicsummer	0.250	0.661	0.46394	0.146640
Post-pandemicsummer	0.263	1.119	0.58597	0.317756

## Data Availability

The data that support the findings of this study are not publicly available due to privacy and ethical restrictions, as they contain sensitive patient information. The anonymized datasets may be available from the corresponding author upon reasonable request and with permission from the relevant institutional ethics committees.

## Supplementary Materials

The following supporting information can be downloaded at: https://www.mdpi.com/article/10.3390/medicina61081398/s1, STROBE Checklist.
